# Dislocation-pipe diffusion in nitride superlattices observed in direct atomic resolution

**DOI:** 10.1038/srep46092

**Published:** 2017-04-06

**Authors:** Magnus Garbrecht, Bivas Saha, Jeremy L. Schroeder, Lars Hultman, Timothy D. Sands

**Affiliations:** 1 Thin Film Physics Division, Department of Physics, Chemistry, and Biology (IFM), Linköping University, SE-581 83 Linköping, Sweden; 2 Department of Materials Science and Engineering, University of California, Berkeley, CA 94720, USA; 3 Bradley Department of Electrical and Computer Engineering and Department of Materials Science and Engineering, Virginia Tech, Blacksburg, VA 24061, USA

## Abstract

Device failure from diffusion short circuits in microelectronic components occurs via thermally induced migration of atoms along high-diffusivity paths: dislocations, grain boundaries, and free surfaces. Even well-annealed single-grain metallic films contain dislocation densities of about 10^14^ m^−2^; hence dislocation-pipe diffusion (DPD) becomes a major contribution at working temperatures. While its theoretical concept was established already in the 1950s and its contribution is commonly measured using indirect tracer, spectroscopy, or electrical methods, no direct observation of DPD at the atomic level has been reported. We present atomically-resolved electron microscopy images of the onset and progression of diffusion along threading dislocations in sequentially annealed nitride metal/semiconductor superlattices, and show that this type of diffusion can be independent of concentration gradients in the system but governed by the reduction of strain fields in the lattice.

Diffusion in crystalline solid-state materials takes place along diverse structural paths. While lattice (or bulk) diffusion (*D*_*l*_) is aided by lattice point defects like vacancies and interstitials, it has been long known that diffusivity in metals is many orders of magnitude higher along dislocations (*D*_*d*_), grain boundaries (*D*_*gb*_), and at free surfaces (*D*_*s*_), forming high-diffusivity paths (cf. Supplementary Figure S1)^1^,^2^,^3^. Knowledge about the structural features along which the diffusion paths are formed is of importance in order to minimize and control the influence on device performance in applications. For instance, kinetic processes limited by grain-boundary and/or dislocation diffusion in materials are known to be diffusion creep, precipitation, coarsening, solute segregation, strain, ageing, grain boundary migration, and sintering^4^,^5^.

In thin film technology in modern electronic devices, high-diffusivity paths play a particular important role since, e.g., structural changes in microelectronic, magnetic storage, and optoelectronic components are governed by short-circuit diffusion^6^. This phenomenon becomes even more relevant for devices based on a multilayer thin-film architecture with individual film thicknesses in the order of the diffusion length at operating temperatures^7^,^8^,^9^. Multilayer systems featuring nitride diffusion barriers have the ability to reduce wear, increase high temperature corrosion- and thermoelectric resistance^10^,^11^,^12^,^13^,^14^,^15^. For instance, we recently reported on the development of single-crystal film epitaxial metal/semiconductor superlattices with low defect densities exhibiting high melting points and mechanical hardness^16^,^17^,^18^,^19^.

However, thin film multilayer structures are at the same time vulnerable due to the possibility of composition- and consequently stress gradients resulting from the growth methods. While state-of-the-art growth techniques and lattice-matched choice of substrate and film materials enable single grain growth of metallic films and hence minimize grain-boundary short-circuit diffusion, dislocations are inevitably present with typical density values for thin metallic films of 10^14^ m^−2^. It is thus obvious that dislocation-pipe diffusion can become the predominant mechanism leading to device failure in thin film multilayer systems at working temperatures. Models to describe the diffusion properties along dislocations assume pipe-like channels along which the diffusion occurs^4^,^20^.

Clouds of impurity atoms near defect lines in alloys, so-called Cottrell atmospheres, can be visualised by atomic probe microscopy^21^. However, besides early work by Volin *et al*. that studied the transfer of vacancies via a dislocation between a void and the surface of an Al foil by transmission electron microscopy (TEM)^22^, and an *in-situ* TEM study by Legros *et al*. from 2008 that demonstrates Si precipitate dissolution via dislocation diffusion in an Al grain at low magnifications^23^, mostly indirect experimental confirmations of the phenomenon of dislocation-pipe diffusion exist^24^. In particular, no direct atomically-resolved observations of the onset and formation of dislocation-pipe diffusion have been reported so far.

## Results and Discussion

Here, we present direct high-resolution TEM observations of the process of pipe diffusion along threading dislocations in recently developed HfN/ScN metal/semiconductor epitaxial multilayers^25^. Dislocations were introduced during the growth by a 7% lattice mismatch of the MgO substrate with the films. Although previous energy-dispersive x-ray spectroscopy (EDS) mapping investigations have demonstrated no measurable bulk diffusion to occur upon annealing for 120 h at 950 °C, we found dislocation-pipe diffusion to take place at isolated dislocations of a sequentially-annealed sample.

**Figure 1:**
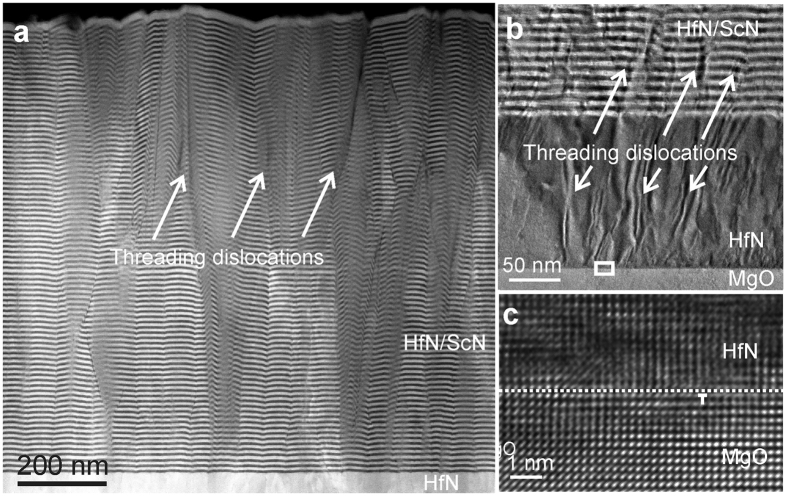
Threading dislocations in a HfN/ScN-metal/semiconductor superlattice introduced by lattice mismatch. (**a**) Low-resolution overview HAADF-STEM micrograph of an as-deposited HfN/ScN superlattice stack grown with a 7% mismatch on MgO with a HfN buffer layer in between. (**b**) TEM micrograph showing threading dislocations originating at the MgO-substrate/HfN-buffer-layer interface and extending throughout the as-deposited superlattice stack. (**c**) Enlarged region of the MgO/HfN interface marked by a rectangle in (**b**) showing edge dislocations at the interface as a result of the lattice mismatch.

Figure 1a and b show overview micrographs of an as-deposited HfN/ScN sample. Threading dislocations originate at the interface between the MgO-substrate and the HfN-buffer layer resulting from the lattice mismatch of nominally 7%. Dislocations typically extend throughout the buffer layer into the metal/semiconductor superlattice. Edge dislocations at the interface between MgO and HfN can be seen in the high-resolution TEM micrograph in Fig. 1c. Summing up the threading dislocations along the superlattice part of the film from many low- magnification micrographs gives an average dislocation distance of ~30 nm corresponding to a density of ~10^15^ m^−2^.

**Figure 2:**
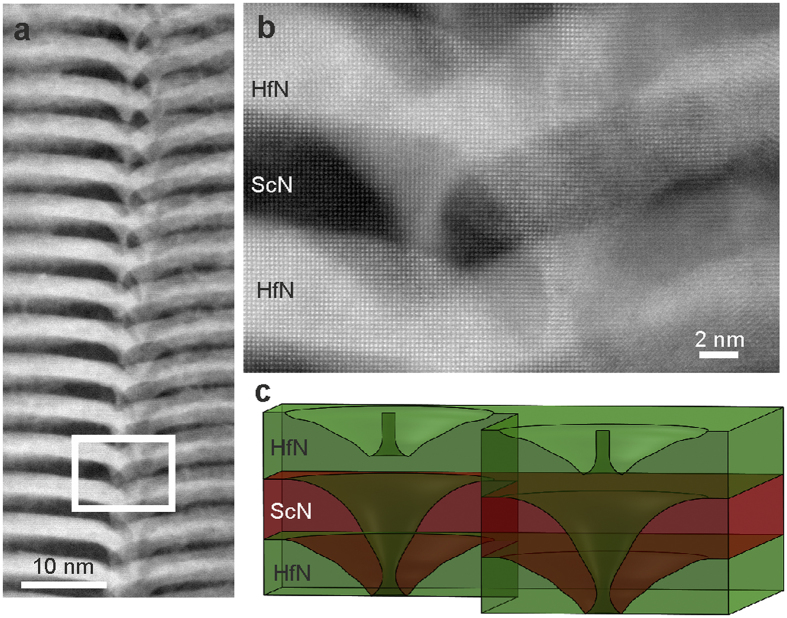
Diffusion of Hf along dislocations in a HfN/ScN superlattice. (**a**) HAADF-STEM micrograph after 120 h annealing at 950 °C shows diffusion of the heavy Hf atoms into the ScN layers forming pipes along the dislocation lines vertically through the superlattice. (**b**) Atomically-resolved STEM micrograph of a region (marked with rectangle in (**a**)) with two dislocations in lateral proximity but different depth of the sample, demonstrating the linear nature of the pipe diffusion process. (**c**) Schematic illustration of the relative positions of the diffusion pipes in (**b**).

The HfN/ScN superlattice samples were annealed at 950 °C for a period of 120 h. Figure 2a shows a high-angle annular dark-field scanning TEM (HAADF-STEM) micrograph of the samples after annealing. The layers are well-separated away from the dislocations. Diffusion of Hf, however, (which appears bright due to the Z-sensitivity of the technique) has clearly taken place along the dislocation lines. An atomically resolved STEM micrograph of the dislocation region in thicker areas of the sample is shown in Fig. 2b. Two threading dislocations can be found in lateral proximity (shown schematically in 2c), but at different depths of the sample, demonstrating the linear “pipe”-character of the diffusion in contrary to planar defects like grain boundaries.

**Figure 3:**
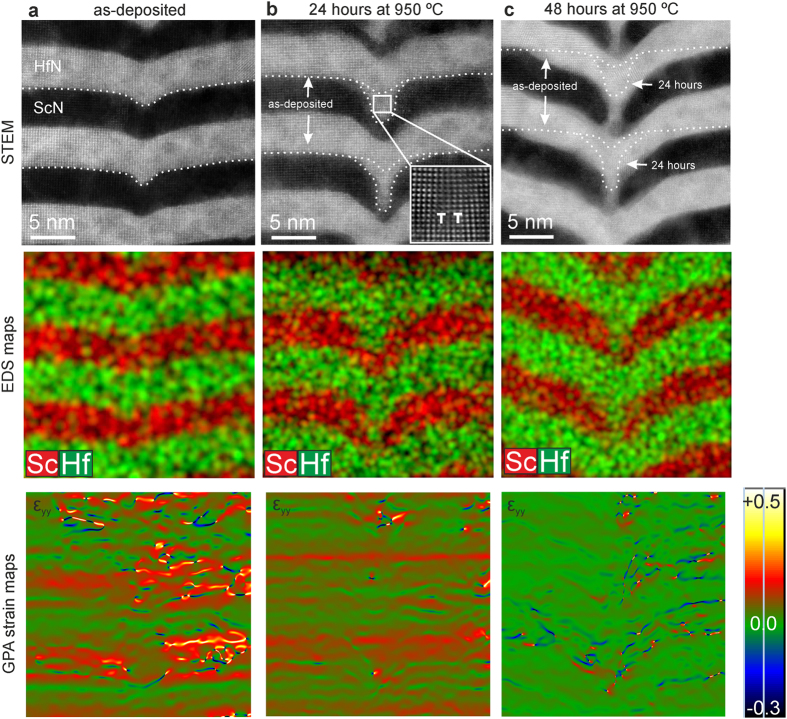
The operation of dislocation-pipe diffusion by strain fields around a threading dislocation line. High-resolution STEM micrographs, corresponding EDS- and strain mapping of the same area of a HfN/ScN superlattice sample as-deposited (**a**), and after annealing for 24 h and 48 h, respectively, at 950 °C (**b**) and (**c**). The onset of Hf diffusion along the dislocation line after deposition is already visible in (**a**). From the change in the shape of the Hf diffusion front after annealing in (**b)**, the diffusion length can be directly measured and an average value calculated. The (contrast enhanced) enlarged region in the inset in (**b**) shows pairs of edge dislocations at the cores of the vertical dislocation line, in the center along which the diffusion occurs. Strain mapping reveals high strain fields around the dislocation line of the as-deposited sample (**a**), that become significantly reduced by the diffusion of Hf after 24 h of annealing (**b**), and relaxes the lattice almost entirely once the pipe formation is completed (**c**). Shown is the y-component of the strain tensor ε_yy_, i.e. parallel to the direction of diffusion. Strain is measured with reference to a lattice region outside the field of view, about 15–20 nm away from the dislocation core. Strain shown in (1/100) %.

In order to quantify the findings above, a sequential annealing series was performed. Figure 3 shows atomically resolved STEM micrographs and corresponding elemental EDS maps together with strain mapping employing geometric phase analysis (shown is the ε_yy_ component of the strain tensor, parallel to the direction of diffusion, see Supplementary Materials online for detailed information) of the threading dislocation line region of the same sample area as-deposited (3a), after 24 h annealing at 950 °C (3b), and after a total of 48 h annealing at 950 °C (3c). The same sample region could be repeatedly identified for the sequential imaging due to a characteristic shape of the sample surface in the area of interest (cf. Supplementary Figure 2). Along the dislocation line in (3a), an onset of diffusion has already taken place during the magnetron-sputter deposition of the layers at elevated temperatures (see methods for growth details). Noticeably, the layers curve towards the substrate while the coherency of the lattice is continuously maintained, unlike as in e.g., low-angle tilt boundaries. After 24 h of annealing, Hf has diffused massively into the ScN along the dislocation line towards the direction of the substrate, however, has not yet reached the respective next neighbouring HfN layers below. Along the path of diffusion, pairs of edge dislocations (enlarged in the contrast enhanced inset in (3b)) become apparent.

After 48 h (3c), the hafnium atoms have formed a pipe-shaped connection between the HfN layers along the vertical threading dislocation line, parting the ScN. The dotted lines in (3b) and (c) serve as guide to the eye to mark the respective shape of the layers as-deposited and after 24 h of annealing. By applying a random-walk law for the diffusion length *d*_*l*_ = *(Dt)*^*1/2*^, we calculate the diffusivity coefficient *D* to 2.34 ·10^−22^ m^2^/s by measuring the average distance *d*_*l*_ the Hf atoms have diffused during *t* = 24 h, (see methods section for detailed description). As can be seen, Hf diffuses along the dislocation pipe towards the substrate direction downwards along a curved frontline, and leaves similarly shaped regions depleted of Hf at the top of the layer behind, which after another 24 h are partly refilled with Hf from the layers above, forming a closed channel around the pipe. Simple geometric considerations give rise to the assumption that the amount of Hf atoms in the curved diffusion front correspond to the amount in the depleted region at the top of the layer, which suggests that the mechanism is of collective nature as assumed by theory^26^. It can be further seen from Fig. 3b and c that the entire metal layers themselves bend downwards in a curved shape under annealing and deform symmetrically around the dislocation line in the direction of the substrate, which in turn suggests a collective motion of the Hf. The diffusion thus has a lateral component within the metallic layer towards the dislocation pipe (2b), and ultimately a vertical one within the pipe once a closed channel throughout the superlattice stack is formed (c).

The bottom row of Fig. 3 shows strain maps generated from the atomically resolved STEM micrographs in the top, showing the y-component of the strain tensor parallel to the direction of diffusion (both x-component and shear do neither show significant strain nor its reduction, as expected from the direction of diffusion). As can be seen from (3a), the region around the threading dislocation line is highly strained initially, while a few tens of nanometres away from it the lattice is almost entirely relaxed (cf. Supplementary Figure 3). During the formation of pipes (3b), the strain becomes significantly lowered and ultimately the lattice is in an almost unstrained state (b). This demonstrates that the dislocation-pipe diffusion takes place as a result of strong strain fields, and serves to reduce those and relax the lattice, which at the same time reduces the chemical potentials of the components. This is an important finding, since it shows that diffusion can be independent of concentration gradients in the superlattices, but still be driven by the thermodynamics of the system.

The directionality of diffusion towards the substrate can be explained by considering the cusping of the layers during growth. By inspecting Fig. 3a, it is clear that the HfN layers tend to wet the ScN, while the ScN layers tend to open the cusp during film growth. The corresponding local increase in layer curvature of the as-grown ScN layer will thus provide preferential Hf diffusion to fill the cusp, as observed in Fig. 3b and c, by virtue of the local capillarity forces at radius of curvature of only a few nm. We also expect there to exist vacancies in excess of thermodynamical equilibrium, which may drive substitutional diffusion, because of trapping from the relatively low temperature of growth and the local surface-geometrical self-shadowing of the deposition flux.

Molecular dynamics simulations have found that intrinsic diffusion without pre-exiting defects along a threading dislocation core dominates at higher temperatures, whereas diffusion along edge dislocations due to vacancies happens much more slowly^27^. Indeed, none of the micrographs displaying edge dislocations in Figs 3b or 2b show Hf diffusion along the direction of the dislocation line.

Experimental diffusion data exist for a few mononitrides^28^, but not for ScN. A single reference can be found for self-diffusion of N in HfN, however no diffusivity coefficient is estimated from that^29^. Moreover, no data exist for this particular metal/semiconductor combination. Previous density functional theory calculations have predicted though that for a HfN/ScN interface, the energetic conditions are more favourable for Hf atoms to diffuse into the ScN than for Sc to diffuse into the HfN layers^30^, which is clearly confirmed by our experimental findings.

**Figure 4:**
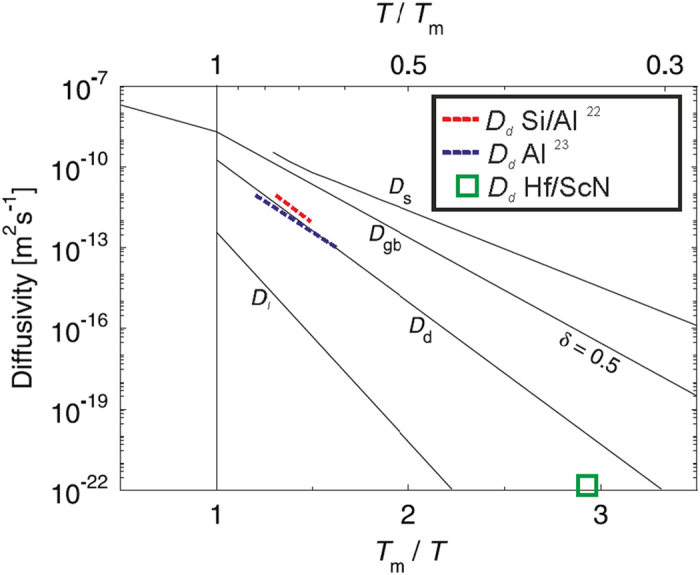
Averaged diffusion spectrum in metals after (5). The diffusivity coefficient of Hf in ScN found in this study is inserted as green rectangle, and the only two other direct experimental finds of dislocation-pipe diffusion from the literature (both done on Al) are inserted for completeness^22^,^23^.

Figure 4 shows averages of typical diffusion spectra for various metals and diffusion paths after^3^. The diffusivity coefficient estimated from our data is inserted as green rectangle, about two orders of magnitude below the average D_d_ values for common metals at that T/T_m_, but still 5–6 orders of magnitude above expected bulk diffusion values. The lower than average D_d_ is expected, since nitride films are used for that very reason as diffusion barriers. For completeness, the data from refs 22 and 23 are inserted as well. Those studies were however performed in Al and close to the melting point, which is at 933 K significantly lower than that of HfN (~3600 K) and hence cannot be directly compared to.

It is important to state that for reasons of actual working temperatures in applications, most nitride films are tested at temperatures around 900 °C, since their purpose is often to prevent diffusion between two other adjacent metal layers from happening in the first place. As a consequence, experimental tracer or electrical studies are commonly performed up to that temperature and confirm the absence of diffusion as a result at these temperatures, e.g. for ZrN and TiN at ~0.25 T_m_^28^.

## Summary and Conclusions

Our results confirm theoretical predictions of diffusion spectra for dislocation-pipe diffusion, and the numerical diffusivity coefficient value extracted from our direct TEM investigations fit well within the general understanding of this diffusion phenomenon. The reduction of strain by the formation of the diffusion pipe shows that the mechanism of dislocation pipe diffusion can be understood independent from concentration gradient considerations in the material, but is governed by the strain fields around the threading dislocation line and growth cusps. Via the reduction of the strain through the diffusion process, the chemical potentials of the Hf thus become reduced as the main thermodynamic condition for diffusion.

The formation of pipes around the dislocation core and at different time steps in an annealing series are the first direct experimental observations of the phenomenon at atomic resolution, and can stimulate more work in the field. Moreover, our extracted diffusion coefficients for the HfN/ScN metal/semiconductor superlattice system are of practical value for applications, since they demonstrate the need for low dislocation densities by lattice-matched choice of substrate and film during growth.

## Methods

### Film growth

The HfN/ScN thin film superlattices were grown on 001-oriented MgO substrates with a reactive dc-magnetron sputtering technique inside a load-locked turbomolecular pumped high vacuum deposition system with a base pressure of 10^−8^ Torr (PVD Products, Inc.). The growth chamber had the capability to accommodate four targets and was equipped with three dc power supplies. The Sc (99.998% purity) and Hf (99.99%) targets had dimensions of 2 in. diameter and 0.25 in. thickness. All depositions were performed with an Ar/N_2_ mixture having 6 sccm of N_2_ and 4 sccm of Ar at a deposition pressure of 5 mTorr. The targets were sputtered in constant power mode, with Sc and Hf power held constant at 200 W. The deposition rates were 4.2 nm/min for ScN and 5.1 nm/min for HfN. The substrates were maintained at 850 °C during deposition, as determined using an infrared pyrometer operated in the wavelength range of 0.8–1.1 μm, together with a thermocouple. The nominal values of the HfN/ScN film structures were: 200 nm HfN buffer/1 μm 6 nm/6 nm HfN/ScN superlattice/20 nm HfN capping layer.

The superlattices were annealed (ramp rate = 20 °C min^−1^) at 950 °C for 24 h, 48 h, and 120 h in a 1.1 Pa (8.5 mTorr) forming gas ambient (5% H_2_:95% N_2_). The custom-designed annealing furnace consisted of a Boralectric tube heater inside a vacuum chamber that was evacuated to < 9.3·10^−5 ^Pa (7·10^−7^ Torr) before continuously flowing 30 sccm forming gas to achieve a pressure of 8.5 mTorr. The 950 °C temperature of the inside heater wall and sample were verified with a dual wavelength pyrometer (CellaTemp PA40; 0.95 and 1.05 μm).

### Electron microscopy

All experiments where conducted with Linköping’s image- and probe-corrected and monochromated FEI Titan^3^ 60–300 microscope equipped with a Gatan Quantum ERS GIF, high-brightness XFEG source, and Super-X EDS detector for ultra-high count rates, operated at 300 kV.

Cross-section TEM samples were prepared conventionally by face-to-face mounting and gluing of two slices of the film/substrate samples followed by mechanical thinning and polishing employing a tripod tool. Thinning to electron transparency was achieved by ion beam milling using a Gatan PIPS ion mill with 5 keV Ar^+^ ions at incidence angles of 8–10° with respect to the sample surface. Subsequent polishing was achieved in a Technoorg Linda ion mill by gradually decreasing the Ar^+^ ion energy from 1 kV to 250 eV to minimize surface amorphisation.

### Calculation of diffusion coefficients

The superlattice stack contains about 90 pairs of HfN/ScN layers, and vertical dislocation lines appear with an average frequency of about 30 nm (c.f. Figs 1 and 2), which allows for statistically meaningful collection of diffusion length data from a few images only. By measuring the distance the Hf has diffused into the ScN during the first 24 h of annealing (c.f. Fig. 3, distance between the dotted lines depicting the as-deposited and 24 h annealed change in shape of the layer front), and employing the simple random walk law for diffusion *d*_*l*_* = (Dt)*^*1/2*^(*d*_*l*_: diffusion length*, D*: Diffusivity Coefficient, *t*: time), a statistical average for *d*_*l*_ of about 4.5 nm/24 h can be estimated, resulting in a diffusivity coefficient of 2.34 ·10^−22^ m^2^/s at the reduced temperature *T*_*m*_*/T (T*_*m, HfN*_ = 3573 K) of 0.34. For comparison, the coefficient for metal atom interdiffusion in TiN/NbN superlattices is reported to be of the same order of magnitude at 900 °C^31^.

It should be noted, that the random walk law for the diffusion length is an approximation that is however justified in the case of the metal/semiconductor superlattices: Since overlaps of diffusion fields from neighbouring dislocations are negligible due to the large average distance of the individual threading dislocations, Type B kinetics according to the classification by Harrison *et al*. occur^32^. However, the usual log *c* versus *z* plot (with *c* being the concentration and *z* the penetration depth) test that is commonly applied, e.g. to distinguish dislocation (log *c* scales linear with *z*) from grain-boundary (log *c* scales linear with *z*^*6/5*^) diffusion tails in tracer studies is not possible for the superlattice systems in this study since it requires measurements over at least two orders of magnitude of *z*, which is inhibited due to the close interlayer distance. More complex diffuser penetration laws^26^,^33^ describing the kinetics in the vicinity of dislocations would require modifications taking the effect of the superlattice sample geometry and layer interfaces into account. An experimental determination would require a large set of data points as function of time, which is far beyond the scope of this study. Hence, the random walk law first order approximation was chosen, which proved a reasonable choice since it yields a diffusivity coefficient for the pipe diffusion in the same order of magnitude as that obtained by other methods on similar superlattices^31^, and those of various other metallic systems (cf. Fig. 4).

### Data processing and software tools

All electron microscopy data were processed using Gatan’s Digital Micrograph. Schematics are drawn in Solid Works and figures were prepared using CorelDRAW.

## Additional Information

**How to cite this article**: Garbrecht, M. *et al*. Dislocation-pipe diffusion in nitride superlattices observed in direct atomic resolution. *Sci. Rep.* **7**, 46092; doi: 10.1038/srep46092 (2017).

**Publisher's note:** Springer Nature remains neutral with regard to jurisdictional claims in published maps and institutional affiliations.

## Supplementary Material

Supplementary Information
