# Supplementary material for: Dislocation-pipe diffusion in nitride superlattices observed in direct atomic resolution
Source: Sci Rep. 2017 Apr 6;7:46092. doi: 10.1038/srep46092 (PMC5382674; doi:10.1038/srep46092)
Supplement: Supplementary Information [file srep46092-s1.pdf]

## Supplementary Information

for

# Dislocation-pipe diffusion in nitride superlattices observed in direct atomic resolution

Magnus Garbrecht, Bivas Saha, Jeremy L. Schroeder, Lars Hultman, and Timothy D. Sands

### Diffusion in solids

In general, the relation $D_l << D_d < D_{gb} < D_s$ holds true for diffusion in metals, as schematically shown in Supplementary Fig. S1. Diffusion profile data for grain- and interphase boundary diffusion as well as lattice (self-) diffusion is compiled for a large number of systems [34, 35]. These data are most commonly based on the radiotracer sectioning technique, as well as various ion- and electron spectroscopies, neutron scattering, or electrical methods like impedance spectroscopy [5]. Model criteria for separating bulk from grain-boundary diffusion in experimental data are applied in practice [36]. However, since practically all metallic crystals contain dislocations, any experimental diffusion profiles will necessarily contain a dislocation contribution that might be negligible near the melting point, but considerably dominant at temperatures below due to the much lower activation enthalpy of dislocation diffusion as compared to that of the lattice. Collections of data using theoretical models in order to separate the dislocation portion from tracer study diffusion profiles can be found [33].

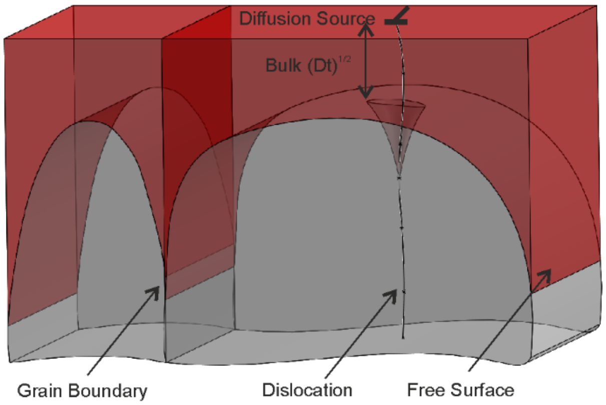


**Supplementary Figure S1. Schematic showing the different modes diffusion can occur in a metallic crystal.** Bulk/lattice diffusion is governed by interstitials and vacancies, and is characterized by its diffusion

length $d_l$ with $d_l = (Dt)^{1/2}$ ($D$: Diffusivity Coefficient, $t$: time). It is typically many orders of magnitude slower than diffusion along linear- or planar crystal defects like dislocations, or grain-boundaries and free surfaces, respectively.

**Ex-situ annealing**

For the annealing series shown in Figure 3, a TEM specimen was prepared from an as-deposited film sample, and subsequently annealed twice by 24 hours each to 950° C. By choice of a characteristically shaped region of interest (cf. Supplementary Fig. S2), the same area of interest could be found again for the scanning imaging and EDS mapping investigations after 24 and 48 hours of annealing.

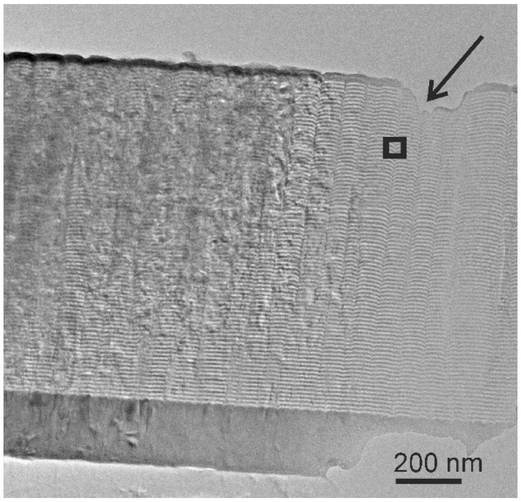


**Supplementary Figure S2. The region of interest of the sequential annealing series.** Due to the characteristic shape of the sample edge in the thin region marked by an arrow, the exact same area for high-resolution STEM imaging and EDS mapping (Fig. 3) could be found after each annealing step. The rectangle marks that area, which can be identified by counting the individual metal and semiconductor layers.

**Geometric phase analysis**

Mapping tensile and compressive strain from atomically resolved images was done employing geometric phase analysis (GPA) using a Digital Micrograph script written by C. Koch [36]. As references, regions of

unstrained lattice further away from the dislocation lines were chosen (see Supplementary Fig. S3 and S4). The strain maps show the y component of the strain tensor ($\varepsilon_{yy}$), which lies vertical to the beam scanning direction, i.e. parallel to the direction of diffusion. The colour scale numbers for the strain maps are given in % of strain divided by 100. Positive numbers mean tensile, and negative numbers compressive strain along the y- direction with respect to unstrained lattice.

The strain maps shown in Figure 3 are calculated by choosing reference regions further away from the dislocation line, and in the HfN layers. This region was chosen to be at the same place for the analysis of the as-deposited and annealed images in Figure 3 to allow for direct comparison. Since the STEM images shown in Figure 3 are cropped to allow for visibility of atomic resolution in print, the reference region lies in outside the area of view as shown in the Supplementary Fig. S4 below.

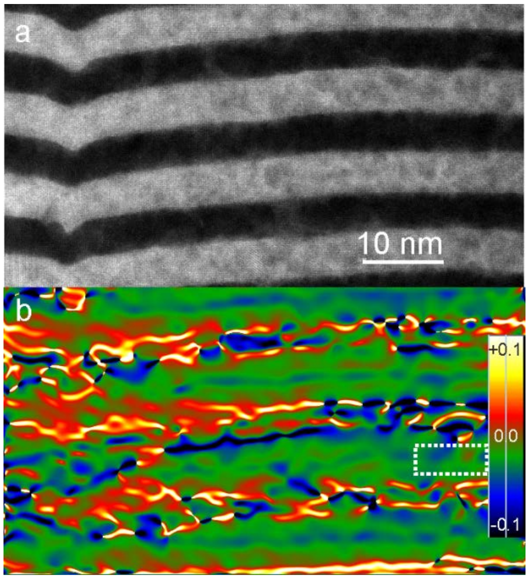


**Supplementary Figure S4. Strain field around the threading dislocations of an as-deposited sample.** Threading dislocations, whose Burgers-Vector lies parallel to their dislocation line in contrast to edge dislocation, are commonly accompanied by strain fields of the lattice. **a**, High-resolution STEM image of an as-deposited sample. **b**, Geometric phase analysis strain mapping reveals a highly strained lattice around the dislocation line, while the strain further away from the dislocation in the lateral direction is significantly lower. Dashed line marks the region the strain is measured relative to.

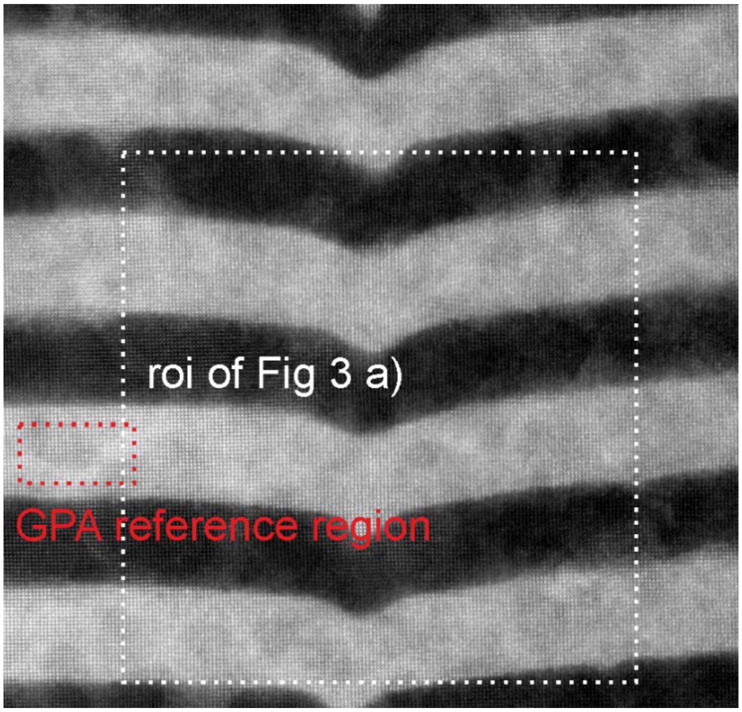


**Supplementary Figure S4. GPA reference region.** The area of the reference region for the GPA maps in figure 3 is marked by a dashed rectangle.

**Dislocations in the superlattices**

Threading dislocations in nitride thin films from various growth techniques are widely studied in the literature including text book examples, and they are always present in epitaxial films on MgO substrate to concentrations observed here. Threading dislocations in cubic systems comprise most commonly both edge and screw components. Those can be distinguished by their Burgers vector which is either directed perpendicular (edge) or parallel (screw) with respect to the dislocation line. Their contrast is easy to spot and distinctive in TEM imaging. In the superlattice film samples in this study, the threading dislocations originate at the interface of the MgO substrate and the HfN buffer from edge dislocation (although not every edge dislocation is accompanied by an upward screwing dislocation), showing their unique contrast best when viewed in bright field with an aperture excluding any but the central (000) beam, as shown below in Supplementary Fig. S5 a. The threading dislocations were analysed by standard TEM and diffraction

methods. Edge dislocations are present at the interface, as is shown in the lattice resolved TEM images of Fig. 1 c and Supplementary Fig. S5 b, marked by circles.

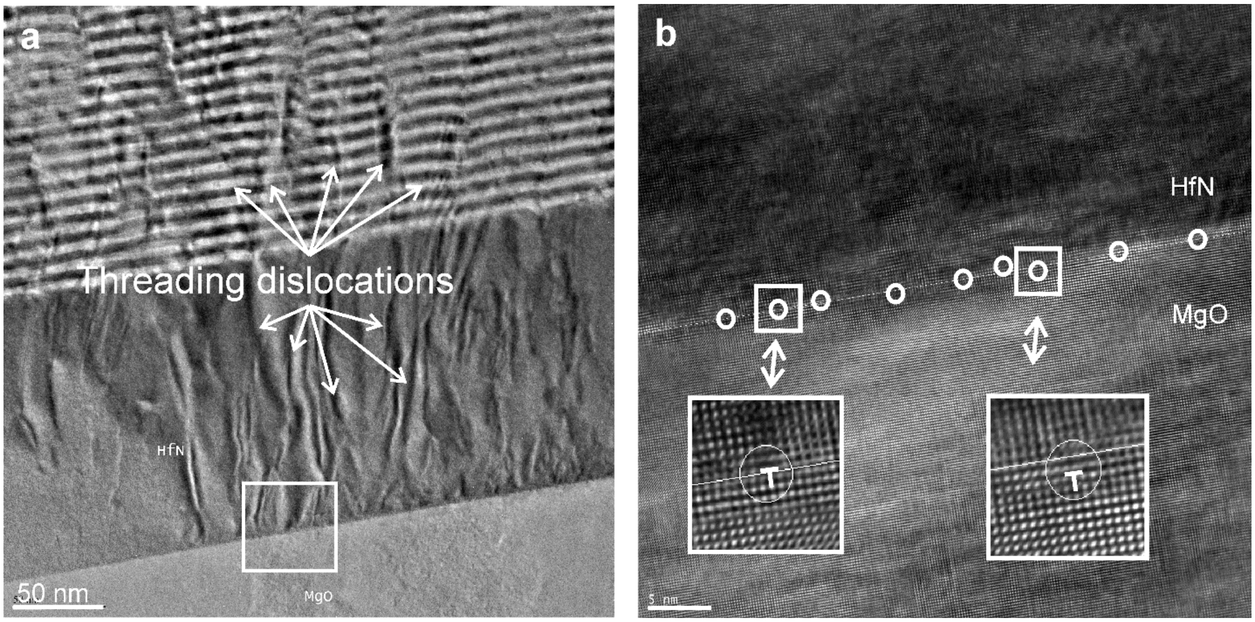


**Supplementary Figure S5. Threading dislocations originating at the substrate film interface. a** TEM image showing the unique contrast of threading dislocations employing an aperture around the (000) reflection at low magnifications. **b** Lattice resolved TEM image showing the presence of edge dislocations at the interface.

The dislocation in the region where the diffusion study has been performed (Fig. 3) has a Burgers vector mainly parallel to its dislocation line and pointing upward towards the free surface, as it is common for threading dislocations. This Burgers vector cannot be imaged in direct lattice contrast in any zone axis accessible by tilt in the TEM given the thin film sample geometry. That would require a close-to perpendicular cut of the TEM lamella which is impossible. Instead, dark-field (DF) analysis was performed as presented in Supplementary Figure S6. Shown are dark-field images employing the (-200) (a) and (002) (b) **g** vectors for comparison with the BF image (c), taken along the [010] axis of the cubic superlattice stack. As can be seen, due to the **g**·**b**= 0 extinction rule the contrast of the threading dislocation vanishes to

black lines for the (-200) reflection, while becoming pronounced in (002) contrast as expected for a burgers vector in parallel with the dislocation line.

Another method for imaging dislocation lines is weak-beam dark-field (WBDF) microscopy, where information is recorded from weakly excited beams. Most commonly, the **g**(3**g**) condition is achieved by beam tilting a distinguished **g** vector onto the optical axis and exciting its respective 3**g** by tilting the sample such that the thin 3**g** Kikuchi band intersects the 3g reflection exactly. An example for a single dislocation in the BF field of view is presented in d, where the dislocation line appears bright in **g**(3**g**) condition. This imaging condition can be matched only locally constricted due to the bending at the thin sample edge. Moreover, the absence of visible Kikuchi lines in the thin parts of the film allows for very faint contrast only. In thick parts of the film however where bending is absent and Kikuchi lines visible, exact adjustment of the **g**(3**g**) conditions is possible revealing pronounced dislocation lines against an almost zero background contrast of the superlattice stack over a large field of view (e), recorded with an exposure time of 40 s.

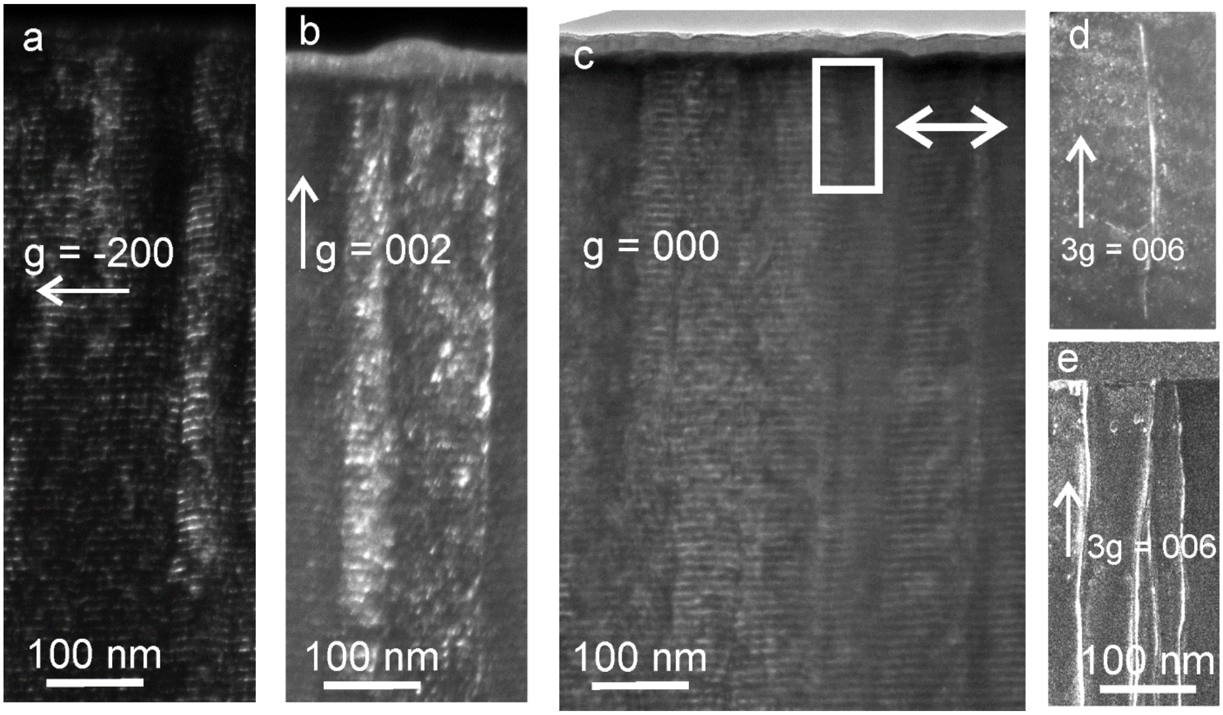

**Supplementary Figure S6. DF and WBDF analysis of the superlattice recorded along [010].** DF images showing vanishing (a), and pronounced (b) contrast of dislocation lines from the threading dislocations shown in the BF overview (c). The line contrast is more pronounced when employing WBDF microscopy at g(3g) condition as shown for both thin (d, 20 s exposure time), and very thick (e, 40 s exposure time) regions.

**Pipe radius and width of stressed lattice regions**

In the original article by Smoluchowski [4] and in subsequent theoretical treatments of the problem, a pipe radius of 0.5 nm is assumed. Figure. 3 c suggests that this value is an underestimation. That is of importance, since theoretically-derived solutions for mean thin layer concentrations assuming a depletable thin-film-like source of atoms show that the contribution of dislocation diffusion is proportional to the total cross-sectional area of dislocations $\pi a2\rho d$, with pipe radius a and dislocation density $\rho d$ [26]. Moreover, as can be seen from Supplementary Figure 3, the strain fields around the dislocation core reach a few tens of nanometres into the lattice, suggesting that the theoretical treatment of the diffusion as function of the pipe diameter might need a modification to address for that.

**References**

34. Kaur, I., Gust, W., Kozma, L. in two volumes: Handbook of Grain and Interphase Boundary Diffusion Data, (Ziegler Press, Stuttgart, 1989).

35. Harrison, L.G. Influence of dislocations on diffusion kinetics in solids with particular reference to the alkali halides. *Trans. Farad. Soc*. **57**, 1191-1199 (1961).

36. Rösner, H., Koch, C., Wilde, G. Strain mapping along Al–Pb interfaces. *Acta Materialia* **58**, 162–172, (2010).
